# Conflict Detection in Moderate Base-Rate Tasks: A Multi-Measure Study

**DOI:** 10.3390/bs13040319

**Published:** 2023-04-07

**Authors:** Jianyong Yang, Zhujing Hu, Dandan Nie, Debiao Zhu

**Affiliations:** School of Psychology, Jiangxi Normal University, Nanchang 330022, China; huzjing@jxnu.edu.cn (Z.H.); niedandan@jxnu.edu.cn (D.N.); zhudebiao@jxnu.edu.cn (D.Z.)

**Keywords:** conflict detection, dual process theory, moderate base rate, storage failure

## Abstract

Empirical studies have found that although humans often rely on heuristic intuition to make stereotypical judgments during extreme base-rate tasks, they can at least detect conflicts between stereotypical and base-rate responses, which supports the dual-processing view of flawless conflict detection. The current study combines the conflict detection paradigm with moderate base-rate tasks of different scales to test the generalization and boundaries of flawless conflict detection. After controlling for possible confounding by the “storage failure” factor, the conflict detection results indicated that reasoners providing stereotypical heuristic responses to conflict problems were slower to respond, less confident in their stereotypical responses, and slower to indicate their reduced confidence than reasoners who answered no-conflict problems. Moreover, none of these differences were affected by different scales. The results suggest that stereotypical reasoners are not blind heuristic performers and that they at least realize that their heuristic responses are not entirely warranted, which supports the argument for flawless conflict detection and extends the boundaries of flawless conflict detection. We discuss the implications of these findings for views of detection, human rationality, and the boundaries of conflict detection.

## 1. Introduction

Human judgment is often biased by erroneous heuristic intuition. Consider the following example [1]:

In a study, 1000 people were tested. Among the participants, there were 995 nurses and 5 doctors. Jake is a randomly chosen participant in this study.

Jake is 34 years old. He lives in a beautiful home in a posh suburb. He is well spoken and very interested in politics. He invests a lot of time in his career.

What is more likely?

(a) Jake is a nurse.
(b) Jake is a doctor.

Based on the sample size of the two groups, a random person would likely be a nurse. However, many people succumb to heuristic intuition and answer that Jake was a doctor based on the stereotypical description, giving rise to the term “base-rate neglect” [2,3]. Much research has shown that similar heuristic intuition can lead people to make biased judgments that violate normative logical or probabilistic considerations [4,5].

### 1.1. Dual Processing Theory and Conflict Detection

The rise and development of dual process theory in human thinking are often linked to the interpretation of reasoning bias. Dual process theory posits that humans have two distinct types of thinking: Type 1 and Type 2 [5,6,7,8,9,10,11,12]. Type 1 (intuitive or heuristic) operates quickly and automatically, whereas Type 2 (deliberate or analytic) operates slowly and requires cognitive resources. Generally, human reasoners tend to base their judgments on fast heuristic intuition rather than deliberative reasoning. Although heuristic intuition may sometimes be beneficial, it also often distorts our judgments when it elicits responses that conflict with normative considerations [5,8].

Although dual process theory provides a compelling explanation for bias, the nature of bias remains controversial among researchers. A crucial issue is whether reasoners who make heuristic responses to conflict problems (referred to as biased reasoners) can detect the conflict between heuristic responses and normative considerations. Two opposing perspectives can be identified here. One is lax conflict detection, represented by the “default-interventionist model”. The default-interventionist model assumes that biased reasoners fail to detect the conflict. The core idea of this model is that people typically rely on fast Type 1 processing to generate a default response when faced with a reasoning problem. One can activate Type 2 processing to intervene and correct Type 1 output [4,8]. However, since Type 2 processing is laborious and complex, people typically do not enable it and stick with the default Type 1 response, leading to bias. The other is flawless conflict detection, represented by the “parallel processing model” [7,12]. The parallel processing model assumes that both minds are activated simultaneously from the beginning of reasoning so that biased reasoners detect the conflict between their heuristic responses and normative considerations. However, the fact that reasoners can detect the conflict does not mean they are always successful in suppressing heuristic responses [13]. When reasoners give heuristic responses to conflict problems, they feel biased but fail to suppress their heuristic intuition. Simply put, the parallel processing model attributes the bias to a failure of inhibition rather than lax conflict detection.

### 1.2. Conflict Detection Paradigm and Related Research

Numerous empirical studies have attempted to define these two detection perspectives through the conflict detection paradigm [1,10,14,15,16]. Participants were asked to solve both conflict and no-conflict problems in this paradigm. The no-conflict problems were constructed by making a minor change to the content of the conflict problems. For example, the no-conflict version of the above base-rate problem would switch the base rate around (e.g., “there were 5 nurses and 995 doctors”). Everything else stays the same. The critical difference between conflict and no-conflict problems is that the heuristic intuition in conflict problems prompts incorrect responses. If biased reasoners at least consider logical or probabilistic rules, it can be predicted that biased reasoners will process the conflict and no-conflict problems differently. Conversely, both versions of the problems should be processed the same way.

The results of conflict detection studies have shown that biased reasoners processed the two versions of the problem differently. Specifically, compared to solving no-conflict problems, biased reasoners showed increased response time [10,14,16,17], increased activation of brain regions associated with conflict detection [18,19], and decreased response confidence [15,20,21] when solving conflict problems. These findings indicate that biased reasoners can detect the conflict and feel that their heuristic responses are problematic. In addition, flawless conflict detection is also observed under severe time pressure and cognitive load [22,23,24,25]. Given that Type 2 processing can be experimentally “knocked out” by limiting participants’ response time or burdening their cognitive resources, this suggests that biased reasoners not only successfully detect the conflict but also do so intuitively based on mere Type 1 processing, leading to a new dual process perspective called the “hybrid” model [6,9,10,23].

### 1.3. Critiques of Conflict Detection Research

However, conflict detection research is also controversial. One of the points of contention relates to the generalization and boundaries of flawless conflict detection. Some researchers have argued that flawless conflict detection may be limited to tasks where the contrast between heuristic and normative responses is obvious [16] or logical or probabilistic rules are relatively simple [14,26]. For example, previous studies have mainly used extreme base-rate tasks (e.g., 995/5) to explore conflict detection in “base-rate neglect”. The base-rate cue is so evident in these tasks that it is easy to draw attention to it. As a result, the flawless conflict detection found in these studies may be specific to the characteristic of extreme base-rate problems.

It should be noted that the correctness or normativity of the base-rate responses in base-rate problems may be questioned [16]. Note that our primary concern is not whether participants are accurate in solving these problems but whether they will detect the inherent conflict. Furthermore, we will adopt more neutral labels such as “base-rate” and “stereotypical” responses in the context of base-rate tasks to minimize misinterpretation.

Nevertheless, conflict detection in moderate base-rate tasks (e.g., 700/300 or 70/30 or 7/3), where the contrast between stereotypical and base-rate responses is relatively obscure, remains unclear. A few studies using moderate base-rate problems have found inconsistent results. Specifically, Pennycook et al. [16] found that conflict detection (as indexed by RT) disappeared when base rates were moderate (70/30). While in a subsequent study, it has found that stereotypical reasoners were able to detect the conflict (as indexed by RT) in moderate base-rate tasks, despite using different scales (700/300) [10]. The latter study also used a novel form of problem presentation called the “rapid response paradigm” and many items. Hence, their results may reflect the greater sensitivity of the “rapid response paradigm” to the RT indicator or just the result of repeated learning due to many items. Moreover, compared with traditional base-rate problems where the base-rate information is presented first, the study also varied the presentation order of base rates and stereotypes (e.g., a stereotypical description was presented first, followed by base-rate information). This simple manipulation may artificially increase the probability of successful conflict detection. Another study combining the “two-response paradigm” with moderate base-rate tasks found that stereotypical reasoners could successfully detect conflict [23], which was reflected in the fact that stereotypical reasoners showed lower response confidence in conflict problems than in no-conflict problems. However, similar to Pennycook et al.’s study [10], Bago and De Neys also varied the presentation order of base rates and stereotypes [23]. In sum, it is difficult to draw decisive conclusions about conflict detection in moderate base-rate problems from these three studies. More research is still needed to test conflict detection in moderate base-rate problems.

### 1.4. Purpose of the Current Study

Previous studies have found inconsistent conflict detection results in moderate base-rate tasks [10,16]. Multiple factors of variation between these studies may affect the results of conflict detection, and these factors of variation also complicate comparisons between these studies. The current study aims to control these discrepancies to more clearly explore conflict detection in moderate base-rate tasks. Two questions will be addressed explicitly in the current study. First, previous studies using moderate base-rate tasks with different scales have found inconsistent results [10,16], and people tend to regard rates with large absolute numbers as having a higher probability of occurrence than equivalent rates with small absolute numbers [7]. Therefore, it is reasonable to suspect that similar base rates but on different scales may affect the stability of conflict detection results. In this study, we explored whether different scales change the results of conflict detection.

Second, previous studies have typically used a single measure to explore conflict detection. Using different measures across studies can increase the generalization and stability of consistent results but will reduce cross-study comparisons between heterogeneous results. The current study will use three measures considered good indicators for conflict detection: response time, response confidence, and confidence response time [21,25,27]. Response time is the amount of time an individual spends processing and evaluating a reasoning problem. If biased reasoners can detect conflict in a conflict problem, then the conflict experience will make them take longer to respond than in a no-conflict problem. Response confidence refers to the level of confidence individuals have in their current response after making that response. If biased reasoners can detect conflict in a conflict problem and feel that their heuristic intuitive response is problematic, then they should show lower confidence after solving the conflict problem than in a no-conflict problem. Confidence response time is the amount of time individuals spend in assigning their confidence levels. Compared with a no-conflict problem, if biased reasoners can detect conflict in a conflict problem, then the sense of conflict will not only reduce their confidence level but also make them spend more time accurately evaluating their confidence level. Using all three measures in the same study, we can comprehensively explore conflict detection in moderate base-rate problems and test the consistency among different measures.

In addition, conflict detection focuses on whether individuals can draw on logical or probabilistic rules to detect the conflict between heuristic and normative responses when needed. Hence, storing logical or probabilistic rules for reasoners is necessary for conflict detection [28]. To avoid the confusion that this “storage failure” factor may cause in conflict detection and to obtain purer conflict detection results, we will use neutral problems to separate the “storage failure” factor [21,29]. The description information in neutral problems will not lead to heuristic responses (see further). Moreover, solving neutral problems relies primarily on familiarity with the relevant logical or probabilistic rules. Consequently, the neutral problems can be used to measure the storage factor.

## 2. Methods

### 2.1. Participants

G*Power 3.1 software [30] was used to calculate the minimum sample size for the experiment. The parameters were set as follows: effect size *f* = 0.25, *α* = 0.05, power (1-*β*) = 0.95, number of groups = 1, number of measurements = 6. The results showed that we needed at least 28 participants. Considering that conflict detection analysis may exclude some subjects, we finally recruited 63 undergraduate students (24 males; *M*_age_ = 20.37, *SD* = 2.16) via flyers at Jiangxi Normal University. All subjects had no history of neurological, psychiatric, or mood disorders. All participants provided written informed consent and received a certain reward after completing the experiment. The study was approved by the Ethics Committee of the School of Psychology, Jiangxi Normal University.

### 2.2. Material

Each participant solved 23 base-rate items, including 2 practice trials, 18 formal trials, and 3 neutral trials. In each trial, the subjects always received a description of both groups in a sample (e.g., “This study contained nurses and writers”), the base-rate information about both groups (e.g., “There were 700 nurses and 300 writers”), and the personality description of an individual randomly selected from the sample (e.g., “Person “A” is creative”). The subjects’ task was to point out which group the randomly selected individual was most likely to belong to.

All problems were adapted from Pennycook et al. [10]. Before the experiment, 25 individuals were asked to rate both groups and the personality description in each question as a match (e.g., what do you think of the match between “nurses” and “creative”? The rating range was 1–7, with 1 for a complete mismatch and 7 for a complete match). A total of 36 ratings were constructed for the 18 formal experimental questions, including 18 high-match ratings and 18 low-match ratings. The results showed that the mean rating score for the high-match group was *M* = 5.90, *SD* = 0.17, and the mean rating score for the low-match group was *M* = 2.30, *SD* = 0.12. There was a significant difference between the two groups, *t* (24) = 86.96, *p* < 0.001, indicating that the personality descriptions in the questions can induce stereotypes of a particular group and thus induce rapid heuristic responses.

Half of the formal problems were conflict items, and the other half were no-conflict items (e.g., nine conflict items and nine no-conflict items). In conflict items, the base-rate and stereotypical information cued different responses (referred to as “base-rate” responses and “stereotypical” responses, respectively). In no-conflict items, the base-rate and stereotypical information cued the same responses (referred to as “base-rate” responses). Table 1 shows examples of conflict, no-conflict, and neutral versions of base-rate tasks. Two different item sets were used. The conflict items in one set were the no-conflict items in the other, and vice-versa. Participants were randomly allocated to one of two item sets.

As already mentioned, we used three scales of moderate base rates [10]: absolutely large (AL) values (e.g., 700/300), absolutely medium (AM) values (e.g., 70/30), and absolutely small (AS) values (e.g., 7/3). We also used three base-rate pairs within the first two scales. In AL, they were 700/300, 710/290, and 720/280; in AM, they were 70/30, 71/29, and 72/28. Considering that the changes in the AS condition might significantly change the range of moderate base rates, only one base-rate pair was used in the AS condition. These slight manipulations made the items less repetitive.

The presentation of all items was based on the rapid-response paradigm [10]. Compared to the traditional presentation form of the base-rate problem (e.g., the base-rate problem at the beginning of Section 1), this paradigm can minimize the influence of reading times and produce a purer measure of reasoning time per se.

### 2.3. Procedure

The experimental tasks were presented using E-Prime 3.0 software. The experimental instructions were presented first. The instructions were as follows:

“Welcome to this experiment. Please read the following instruction carefully.

A large number of studies were conducted in a big research project.

In each study, there are participants from two populations and basic information about the composition of the population.

In each study, one participant was randomly selected from the sample, and you will see a characteristic description of that participant. You need to point out which population the randomly selected participant is more likely to belong to.

An example of a complete question is as follows:

This study contained lawyers and engineers

There were 995 lawyers and 5 engineers

Person “L” is argumentative

Is person “L” more likely to be:

◯ A lawyer ◯ A engineer

Please answer the questions as quickly and accurately as possible.

If you understand the instructions, please click “NEXT” to enter the exercise.

After the instruction, participants were presented with two practice problems to familiarize them with the procedure. Then, participants had to solve 18 main experimental items presented randomly. Finally, the neutral items were given as the last three trials in the whole experiment so that they would not prime participants’ subsequent responses.

Each problem started with a fixation cross for 1000 ms. After the fixation cross disappeared, the sentence which specified the two groups appeared for 2000 ms. Then the base-rate information appeared, for another 2000 ms, while the first sentence remained on the screen. Finally, the stereotypical information appeared together with the question and two response alternatives. Once all the parts were presented, participants could select their answers by clicking on them, and the response time indicator collected exactly the time of this interface. The position of the response alternatives was randomly determined for each item. After making a response, participants were presented with a new interface. Participants were asked to indicate confidence in their responses by clicking a number on a scale that increased in gradations of 5% from 0 (not at all confident) to 100% (completely confident). The time participants spent on this confidence rating interface was the confidence response time.

## 3. Results

### 3.1. The Proportion of Base-Rate Response Choices

**Figure 1:**
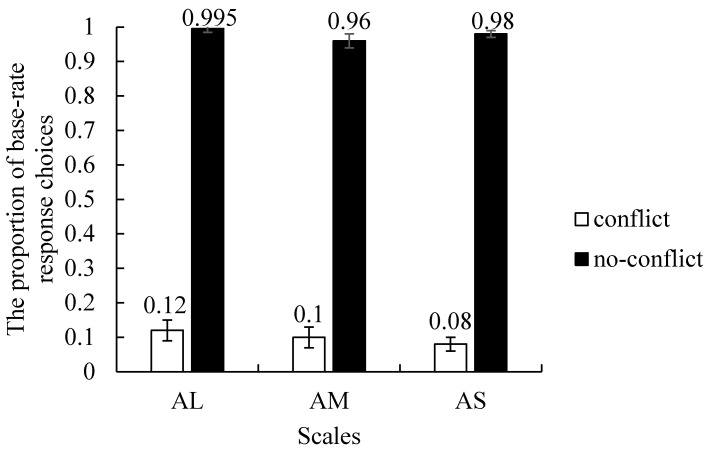
The proportion of base-rate response choices for each condition. Error bars are standard errors.

We ran a 2 (Conflict: conflict, no-conflict) × 3 (Scales: AL, AM, AS) within-subject analysis of variance (ANOVA) on the proportion of base-rate response choices. As Figure 1 shows, there was a main effect of the Conflict factor, with more base-rate responses for no-conflict (*M* = 0.98, *SE* = 0.01) than conflict (*M* = 0.10, *SE* = 0.02) problems, *F* (1, 62) = 1719.47, *p* < 0.001, *η*^2^*_p_* = 0.97, which replicates the classic “base-rate neglect” effect [1,2,3]. The main effect of the Scales factor was not significant, *F* (2, 124) = 2.0, *p* = 0.14 *η*^2^*_p_* = 0.03, and neither was a Conflict × Scales interaction, *F* (2, 124) < 1, *p* = 0.55, *η*^2^*_p_* = 0.01.

### 3.2. Neutral Items Analysis

We first made statistics on each participant’s accuracy in neutral problems to exclude possible confusion about conflict detection caused by the “storage failure” factor. It turned out that except for one person who gave incorrect responses to all three neutral problems, everyone responded correctly to at least one neutral problem (*N_A_*_CC (0.33)_ = 14 (22%), *N*_ACC (0.67)_ = 23 (37%), *N*_ACC (1)_ = 25 (40%)), indicating that almost all subjects have minimal storage of the logical or probabilistic rules.

Previous studies have suggested that the extent to which logical or probabilistic rules are stored is an important factor affecting conflict detection [21,29]. To avoid artificially increasing the likelihood of successful conflict detection, we included all individuals with minimal storage of the logical or probabilistic rules in the subsequent conflict detection analysis. That is, only one person was excluded from the conflict detection analysis.

### 3.3. Conflict Detection Analysis

In line with previous work, the conflict detection analysis focused on the difference between incorrectly solved conflict trials and correctly solved no-conflict trials [10,21,27,31], with correctly solved conflict trials not being analyzed. Meanwhile, the rare trials in which no-conflict problems were solved incorrectly were discarded. In addition, participants who did not give any stereotypical responses to conflict problems (*n* = 1) were also dropped from the analysis.

#### 3.3.1. Response Time

**Figure 2:**
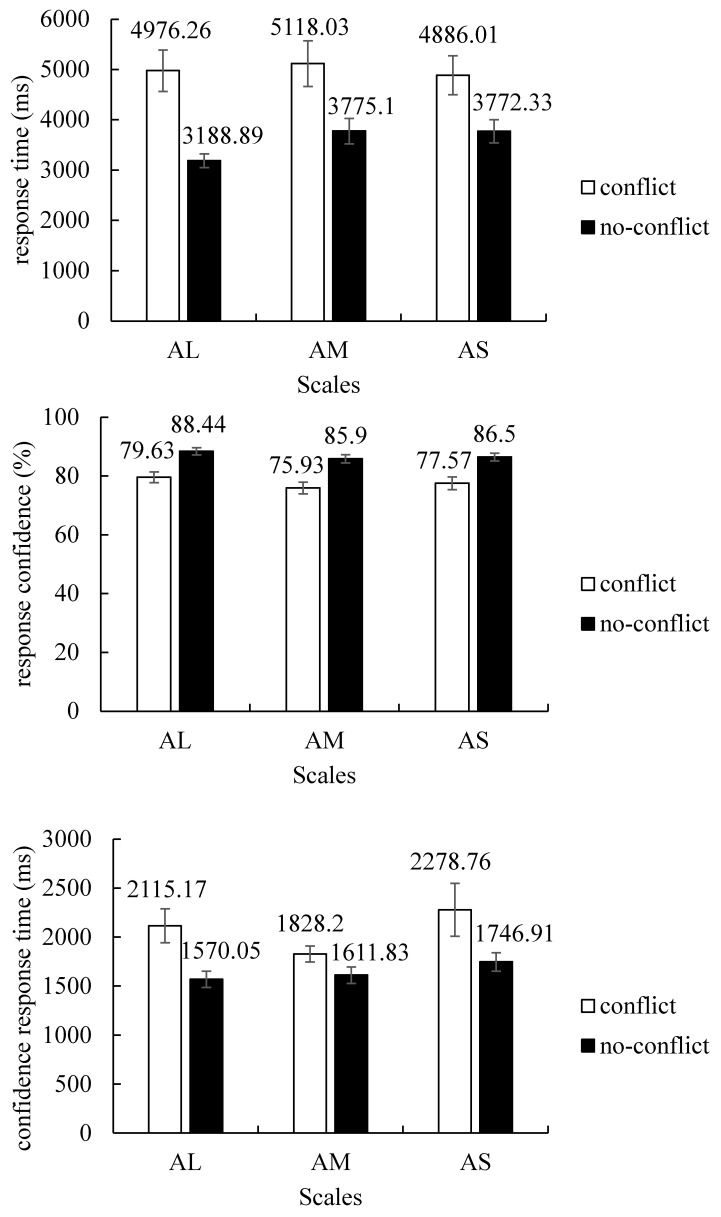
Response time (ms), response confidence (%), and confidence response time (ms) for “stereotypical” responses on the conflict problems and “base-rate” responses on the no-conflict problems in the AL, AM, and AS conditions. Error bars are standard errors.

A repeated-measures ANOVA was run on response time, with Conflict (conflict, no-conflict) and Scales (AL, AM, AS) as independent variables. The top panel of Figure 2 shows the results. A main effect of Conflict factor (*F* (1, 60) = 16.82, *p* < 0.001, *η*^2^*_p_* = 0.22) indicated that, although reasoners failed to give “base-rate” responses to conflict problems, they were indeed spending more time (*M* = 4993.43, *SE* = 387.35) responding than when answering no-conflict problems (*M* = 3578.77, *SE* = 183.36). This suggests that biased reasoners can detect the conflict despite their ultimately “stereotypical” responses. In contrast, we found no significant effect of Scales factor, *F* (2, 120) = 2.70, *p* = 0.07, *η*^2^*_p_* = 0.04, and, critically, no significant interaction between Conflict and Scales, *F* (2, 120) = 1.93, *p* = 0.15, *η*^2^*_p_* = 0.03.

#### 3.3.2. Response Confidence

We then performed a 2 (Conflict: conflict, no-conflict) × 3 (Scales: AL, AM, AS) within-subject ANOVA on response confidence. The middle panel of Figure 2 shows the results. First, a main effect of Conflict factor (*F* (1, 60) = 39.96, *p* < 0.001, *η*^2^*_p_* = 0.40) indicated that when participants gave “stereotypical” responses to conflict problems, their confidence (*M* = 77.71, *SE* = 1.84) was significantly lower than the confidence of reasoners who correctly solved the no-conflict versions (*M* = 86.95, *SE* = 1.17). In line with previous confidence findings [15,21], this indicates that “stereotypical” reasoners are not simply oblivious to the conflict but indeed feel that their “stereotypical” responses are not fully warranted. Moreover, we also found a main effect of Scales factor, *F* (2, 120) = 6.57, *p* = 0.002, *η*^2^*_p_* = 0.10. A post hoc test revealed that participants seemed to have greater response confidence after solving absolutely large-value problems (*M* = 84.04, *SE* = 1.37) compared to absolutely medium-value (*M* = 80.92, *SE* = 1.47) and small-value problems (*M* = 82.04, *SE* = 1.50). However, as with the response time, the critical Conflict × Scales interaction was nonsignificant, *F* (2, 120) = 0.29, *p* = 0.75, *η*^2^*_p_* = 0.01. This confirms that the conflict detection process operates independently of the base-rate scales.

#### 3.3.3. Confidence Response Time

Similar to response time analysis, the mean values of confidence response time were submitted to a 2 (Conflict: conflict, no-conflict) × 3 (Scales: AL, AM, AS) repeated-measures ANOVA. The bottom panel of Figure 2 shows the results. A main effect of Conflict factor was found, *F* (1, 60) = 11.92, *p* = 0.001, *η*^2^*_p_* = 0.17, indicating that participants were significantly slower to report their confidence on conflict problems (*M* = 2074.04, *SE* = 130.03) compared to on no-conflict problems (*M* = 1642.93, *SE* = 72.18). This again suggests that reasoners can detect the conflict between their “stereotypical” response and the base-rate one, which is in line with previous studies focusing on confidence response time as a kind of conflict detection measure [25]. At the same time, neither the effect of the Scales factor (*F* (2, 120) = 2.87, *p* = 0.06, *η*^2^*_p_* = 0.05) nor the Conflict × Scales interaction (*F* (2, 120) = 0.97, *p* = 0.38, *η*^2^*_p_* = 0.02) was significant. The lack of significant interaction suggests that the manipulation of Scales has no significant impact on conflict detection.

#### 3.3.4. Correlation Analysis

We used three measures in the current study to increase the stability of the results. Nevertheless, one might want to know whether specific measures are more relevant than others. Therefore, we next analyzed the correlation between these three measures. Previous studies have suggested that the size of the conflict detection effect could be obtained by calculating an individual’s average difference between incorrectly solved conflict problems and correctly solved no-conflict problems [21,27,31]. Since our conflict detection results show that conflict detection is independent of the Scale factor, we combined the different scales in a composite to calculate the size of the conflict detection effect for each of our three measures. It is of note that the values of response confidence were recoded such that a larger value implies a larger detection effect size.

The correlation analysis showed a strong correlation between response time and response confidence index, *r* = 0.42, *p* = 0.001. Response time also significantly correlated with confidence response time, *r* = 0.60, *p* < 0.001, and the correlation between response confidence and confidence response time had also reached significance, *r* = 0.31, *p* = 0.015. This suggests that the three measures are strongly related, and the conflict detection results show high consistency.

## 4. Discussion

Previous conflict detection studies have indicated that people can detect the conflict between stereotypical and base-rate responses in “base-rate neglect” [1,5,20,21]. However, these findings mainly used extreme base-rate problems, leaving the question of whether conflict detection remains successful in moderate base-rate tasks. The current study uses three measures to explore the conflict detection process in moderate base-rate tasks of different scales while minimizing the potential confusion caused by the “storage failure” factor. As a result, and consistent with decades of reasoning and decision-making research [1,2,15,21], the proportion of base-rate response choices showed that individuals were typically biased and failed to select normative base-rate responses in conflict problems. However, our three measures indicated that, despite this resounding bias, reasoners detected that their intuitively stereotypical responses were not entirely warranted. Specifically, reasoners who gave intuitive but illogically stereotypical responses to conflict problems were slower to respond, less confident in their stereotypical responses, and slower to indicate their reduced confidence than reasoners who answered no-conflict problems. This confirms and extends previous work showing that reasoners are not entirely oblivious to their intuitive but problematic responses [1,10,15,23,32].

Although our research revealed that different scales might affect the relative size of people’s response confidence, the key finding was that all three measures showed that conflict detection would not be affected by different scales of similar base rates. This implies that the inconsistency between the results of the two previous studies [10,16] is not due to the use of different scales. In addition, the current study also presented fewer items than Pennycook et al. [10] (18 vs. 132), which could reduce confusion from training. Given that conflict detection results in the current study show a consistent pattern with the findings of Pennycook et al. [10], one possible explanation for the inconsistent results of the two previous studies is that the “rapid response paradigm” used in the current research and in Pennycook et al.’s research may be more sensitive to the changes in response time. This paradigm can minimize the confusion caused by semantic reading and comprehension of the response time measurement to obtain a purer response time index of conflict detection. Moreover, the high degree of correlation and consistency among the three measures also provides some support for this argument.

The data from this study suggested that stereotypical reasoners could detect the conflict between the stereotypical and base-rate responses in moderate base-rate problems. To be clear, although the default-interventionist model does not predict this successful conflict detection, it fits well with both the parallel [7] and hybrid models [9,10,23]. Indeed, the proponents of the parallel and hybrid models all predict that stereotypical reasoners can detect a conflict. The key difference is that the parallel model believes that the conflict results from the parallel processing of intuitive Type 1 thinking and deliberate Type 2 thinking. In contrast, the hybrid model proposes that the conflict results from two competing Type 1 intuitions, often called heuristic intuition and logical intuition. Note that although our research cannot directly clarify the source of the conflict, the analysis of neutral problems reveals that almost all subjects have minimum storage of logical or probabilistic rules, which indicates that educated adults appear to have sufficient knowledge of logic or probability and may automatically activate the relevant knowledge to a certain extent. Consequently, we believe the current results are easier to reconcile with the hybrid model. However, the present work mainly focuses on whether stereotypical reasoners can detect conflict in moderate base-rate problems, so it is not enough to accurately locate the source of conflict. Future research needs to test the source of conflict directly.

The current study also contributes to the discussion of human rationality. Specifically, the lax detection view argues that reasoning bias is mainly due to failed conflict detection. That is, people just blindly follow heuristic intuition without considering logical or probabilistic rules at all. This view holds that the divergence between biased and unbiased reasoners occurs early in reasoning. Thus, lax detection holds a relatively pessimistic attitude toward human rationality. In contrast, the flawless detection view holds that biased reasoners can detect conflict, and bias originates from the failure of inhibition. This view holds that biased and unbiased individuals are similar in the early reasoning stage. The difference lies in the success or failure of suppressing heuristics at the later reasoning stage. The flawless detection view is relatively optimistic about human rationality. Previous studies have found conflict detection to be flawless mainly in extreme base-rate tasks, which limits the scope of optimistic rationality to tasks with simple logical or probabilistic rules. The current study found that stereotypical reasoners could also detect conflict in moderate base-rate tasks with high logical or probabilistic complexity, showing flawless conflict detection, which expands the scope of optimistic rationality and implies that human rationality is relatively optimistic in a wider range of situations.

It needs to be clarified that although all three measures in the current study suggest that stereotypical reasoners can successfully detect—at least at the group level—the conflict in moderate base-rate tasks, the current study does not argue against possible individual differences in the conflict detection process. Several studies have found that although people have successful conflict detection at the group level, there is still a proportion of reasoners who fail to detect the conflict (e.g., the response time/response confidence/confidence response time of individuals incorrectly solving conflict problems is lower/higher/lower than that of correctly solving no-conflict problems) at the individual level [10,21,27,31]. In short, the current study aims to provide an in-depth and clear exploration of conflict detection in moderate base-rate tasks through multiple measures, focusing primarily on modal reasoner and group-level effects. Future research could build on the current findings to further explore individual differences in conflict detection.

Note that our research also has some implications for exploring the boundary conditions of the two conflict detection perspectives. First, we should remember that it is almost impossible for people to have flawless conflict detection about every problem they need to solve in life. Task difficulty or complexity is an important factor affecting conflict detection [33]. Some researchers have suggested that more difficult tasks requiring more complex logical or probabilistic computations will not lead to successful conflict detection [6,14]. The current study has found that stereotypical reasoners can successfully detect—at least at the group level—the conflict between the stereotypical and base-rate responses when facing the conflict versions of the moderate base-rate problems. On the one hand, this suggests that the threshold required for successful conflict detection during base-rate tasks is lower than 70%. More research is required to define this effect’s boundary base-rate values. On the other hand, it is a convenient and practical, but relatively conservative method, which can be used to explore the boundary conditions by gradually changing the base-rate values. Additionally, using more realistic and complex reasoning scenarios from daily life may also be a good way to test boundary conditions. In addition to qualitatively measuring the success or failure of conflict detection on a given task difficulty or complexity, one can also test for variation in the size of the conflict detection effect by manipulating different task difficulties or complexities. It can be expected that the size of the conflict detection effect tends to decrease as task difficulty or complexity increases. Thus, researchers can predict boundary conditions by testing the magnitude of the decrease in the size of the conflict detection effect.

Finally, we need to point out the limitations of the current study. Previous studies using response time indicators have found inconsistent conflict detection results [10,16]. One possible reason is that the measurement of the response time indicator is not pure enough. To obtain pure conflict detection measures, the current study used neutral items to eliminate the interference of the “storage failure” factor [21,29] and the rapid-response paradigm to minimize the interference of reading time [10]. In addition, the current study also used a within-subject design to exclude the effect of individual differences on the three measures. The within-subject design, however, was subject to a training effect. Frequent trial switching may have artificially increased the likelihood of successful conflict detection. Although the current study used a few items to minimize the effect of training, the most critical approach to address this issue may be the between-subject test, especially the between-subject test where only one question is presented in each condition. Therefore, future studies may consider using the between-subject test to replicate the current study. Combining the between-subject and within-subject test results, one can have a more comprehensive and clearer understanding of conflict detection in the moderate base-rate task.

## 5. Conclusions

Overall, the current study found that although reasoners ended up with stereotypical responses in moderate base-rate tasks, they were not blind heuristic performers and could at least detect the conflict between stereotypical responses and base-rate responses. This suggests that flawless conflict detection is not task-specific, extending the boundaries of flawless conflict detection. It must be emphasized that the current results do not imply that there is no boundary for flawless conflict detection, and more research is still needed to explore the specific boundary conditions.

## Figures and Tables

**Table 1:** Examples of conflict, no-conflict, and neutral versions of base-rate tasks.

Conflict Version		No-Conflict Version		Neutral Version	
This study contained nurses and writers		This study contained farmers and models		This study contained sailors and journalists	
There were 700 nurses and 300 writers		There were 700 farmers and 300 models		There were 700 sailors and 300 journalists	
Person “A” is creative		Person “B” is diligent		Person “D” has dark hair	
Is person “A” more likely to be:		Is person “B” more likely to be:		Is person “D” more likely to be:	
◯ A nurse	◯ A writer	◯ A farmer	◯ A model	◯ A sailor	◯ A journalist

## Data Availability

The datasets generated and analyzed during the current study are available from the corresponding author on reasonable request.
